# Complementary spin transistor using a quantum well channel

**DOI:** 10.1038/srep46671

**Published:** 2017-04-20

**Authors:** Youn Ho Park, Jun Woo Choi, Hyung-jun Kim, Joonyeon Chang, Suk Hee Han, Heon-Jin Choi, Hyun Cheol Koo

**Affiliations:** 1 Center for Spintronics, Korea Institute of Science and Technology, Seoul 02792, Korea; 2 Departement of Materials Science and Engineering, Yonsei University, Seoul 03722, Korea; 3 KU-KIST Graduate School of Converging Science and Technology, Korea University, Seoul 02481, Korea

## Abstract

In order to utilize the spin field effect transistor in logic applications, the development of two types of complementary transistors, which play roles of the *n*- and *p*-type conventional charge transistors, is an essential prerequisite. In this research, we demonstrate complementary spin transistors consisting of two types of devices, namely parallel and antiparallel spin transistors using InAs based quantum well channels and exchange-biased ferromagnetic electrodes. In these spin transistors, the magnetization directions of the source and drain electrodes are parallel or antiparallel, respectively, depending on the exchange bias field direction. Using this scheme, we also realize a complementary logic operation purely with spin transistors controlled by the gate voltage, without any additional *n*- or *p*-channel transistor.

The metal oxide semiconductor (MOS) transistor is the most important component in the semiconductor circuits due to its low-power and high-speed switching operation. In a conventional MOS logic device, two types of transistors, *n*-type MOS (*n*-MOS) and *p*-type MOS (*p*-MOS) transistors, are required for the complementary operation. Generally, an *n*-MOS transistor consists of a *p*-type substrate with *n*-type source and drain electrodes, while a *p*-MOS transistor consists of an *n*-type substrate with *p*-type source and drain electrodes. A complicated doping process is required for fabricating *n*-type and *p*-type devices. For an *n*-MOS (*p*-MOS) transistor, the device is on when the gate voltage is in the high (low) state. Thus, the two transistors are operating in complementary mode, so the logic operation using these transistors is known as complementary MOS (CMOS) logic^1^. The use of a complementary scheme comprising both types of transistors is an essential prerequisite for the construction of a low power logic gate. The spin field effect transistor (spin-FET) potentially has low-power and high-speed operation, so it should be excellent candidate for the logic circuits. In the classical Datta-Das spin-FET^2^,^3^ the spin orientation is controlled by applying an electric field. The operation of this device has been experimentally demonstrated using a quantum well channel and ferromagnetic electrodes. The ferromagnetic electrodes play roles of the drain and source electrodes, instead of an *n*- or *p*- doped semiconductor layers. Therefore, the doping process used to fabricate *n*-MOS and *p*-MOS cannot be utilized, so that the conventional complementary scheme is not possible in the classic Datta-Das type spin-FET.

Spin-based logic devices based on spin transport phenomena other than spin-FET are also being developed. A full-adder^4^ and a magnetic switch^5^ have been realized by using magnetic tunneling junction and current driven switching. In addition, the logic operations were experimentally illustrated using the spin Hall effect^4^ and magnetic field control^5^. Dery *et al*.^6^ and Behin-Aein *et al*.^7^ suggested logic devices using spin injection and accumulation. Also, Kunihashi *et al*.^8^ proposed the complementary operation using channel direction dependence of spin-orbit interaction. While many types of spin logic devices have been reported, there has been no experimental demonstration of complementary operation due to the absence of two complementary devices such as an *n*-MOS and a *p*-MOS.

## Results

### Operation mechanism of complementary spin transistors

In this research, we experimentally demonstrate the complementary transistors consisting of two types of spin-FET. The main idea is that *n*- and *p*-MOS transistors are substituted by parallel- and antiparallel types of spin-FET (P-ST and AP-ST), respectively^9^,^10^. In this research, we basically adopt the operation mechanism of spin-FET. The key function of the spin-FET is the gate modulation of the spin orientation which is initially determined by the magnetization direction of the ferromagnetic source. The fast moving electrons (*k*_*x*_) in an electric field (*E*_z_) induce an effective magnetic field (*B*_R*y*_) known as the Rashba field^11^,^12^,^13^. Inside the channel, the injected spins precess around the axis of the Rashba field which is controlled by an external gate voltage^3^,^13^,^14^,^15^,^16^,^17^,^18^. When the spins arriving at the drain are parallel (antiparallel) to the magnetization of the drain, the spin-FET is in the ON (OFF) state.

**Figure 1:**
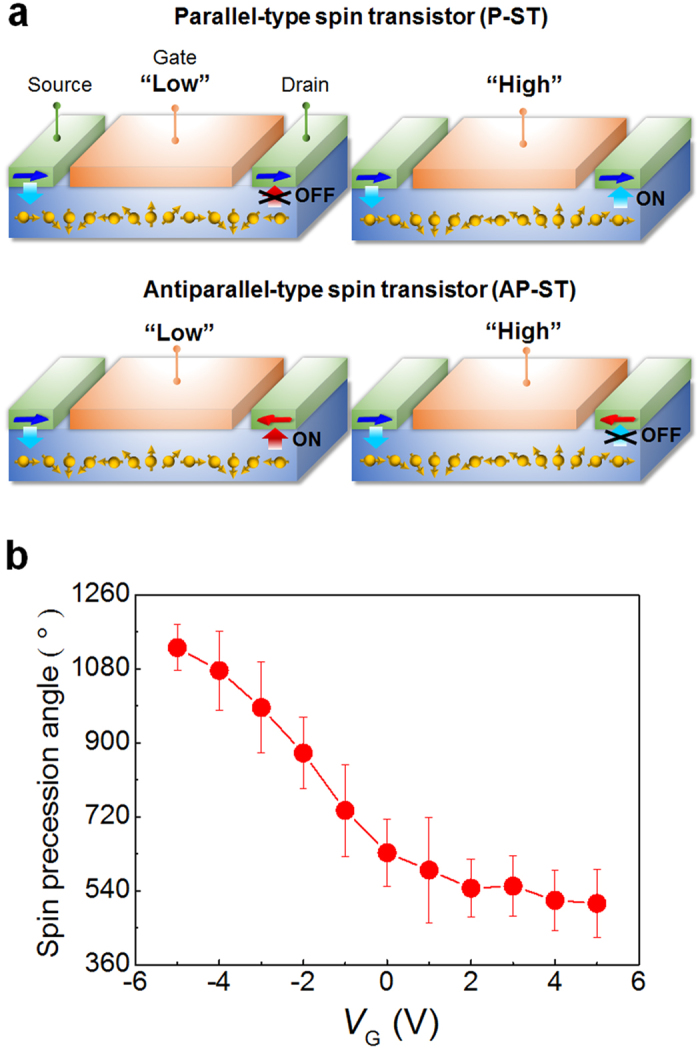
Design of spin-based complementary operation. (**a**) Operation of parallel-type (P-ST) and antiparallel-type spin transistors (AP-ST). The P-ST is on state for *V*_G_ = “Low” and the AP-ST is on state for *V*_G_ = “High”. Spin vectors precess around the Rashba axis of the channel. (**b**) Gate voltage dependence of spin precession angle for a channel length *L* = 1.6 μm. *T* = 1.8 K. Gate voltage controls the strength of Rashba effect and spin precession angle. Error bars represent standard deviations.

Figure 1(a) shows the schematic structures of a parallel-type spin transistor (P-ST) and an antiparallel-type spin transistor (AP-ST). In these transistors, the source and drain electrons are made of ferromagnetic materials. While the magnetization directions of the source and drain electrodes are parallel in the P-ST, the magnetization directions of these two electrodes are antiparallel in the AP-ST. The channel length is carefully selected so that the spin precession angles, Δθ, are *m* × 180° (*m* odd) for *V*_G_ = “Low” and *m* × 180° (*m* even) for *V*_G_ = “High”. As shown in the P-ST of Fig. 1(a), when *V*_G_ is “Low” or Δθ = *m* × 180° (*m* odd), the spins arriving at the drain and the magnetization direction of the drain are antiparallel, so the P-ST is OFF. In the AP-ST for Δθ = *m* × 180° (*m* odd), these two vectors are parallel, so the AP-ST is ON. When *V*_G_ is “High” or Δθ = *m* × 180° (*m* even) in the P-ST and AP-ST, the spins arriving at drain are parallel and antiparallel to the magnetization of the drain, respectively, so only the P-ST is ON. Thus, the P-ST is ON for *V*_G_ = “High” and AP-ST is ON for *V*_G_ = “Low”. This scheme is analogous to a conventional CMOS design in which only *n*-MOS (*p*-MOS) transistor is on when the gate is in the high (low) state. In our complementary transistors, the complicated doping processes for fabricating *n*-MOS and *p*-MOS are not necessary.

In our transistor channel, an InAs-based quantum well structure is utilized^3^,^19^,^20^. The intrinsic electric field is induced by the structural asymmetry in the quantum well. The fast moving electron with an intrinsic electric field produces the Rashba field which drives spin precession. For a channel length *L* = 1.6 μm, the spin precession angle (Δθ) as a function of gate voltage (*V*_G_) is shown in Fig. 1(b). The accumulated spin precession angle inside the channel can be expressed as^2^,^3^, Δθ = 2 m^*^α*L*/*ħ*^2^, where m^*^ and *L* are the effective mass and the length of the channel, respectively. The gate voltage determines the Rashba parameter (α) and spin precession angle so the spin transistor operation is possible. From the channel length dependence of spin transport experiment^19^, a spin diffusion length of 1.8 μm is obtained.

### Magnetization control of source and drain

**Figure 2:**
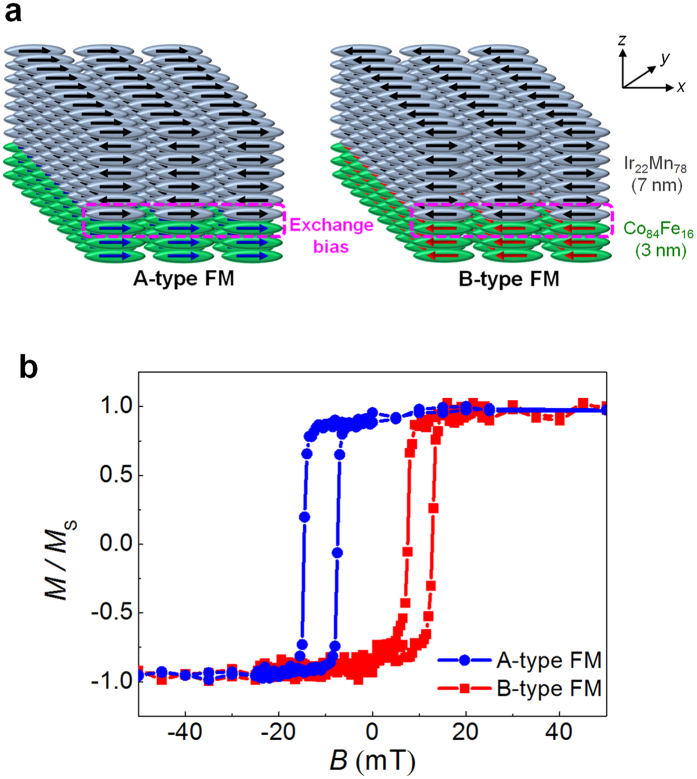
Magnetization control of source and drain. (**a**) Exchange biased source and drain. An exchange bias field is induced from Co_84_Fe_16_/Ir_22_Mn_78_ bilayers. (**b**) Magnetization curves. The shift direction of magnetization curves are controlled by the bias field during the sputtering process.

In our channel structure, the Rashba field arises along the *y*-axis, so the magnetization direction of source and drain should be along the *x*- or *z*-axis, i.e. perpendicular to the Rashba field (*B*_R*y*_), to induce spin precession. In this experiment, we choose the ferromagnetic electrodes (FM) with magnetization along the *x* axis. The lateral sizes of FMs are 0.5 μm × 15 μm and are 0.8 μm × 15 μm, respectively. Since the shape anisotropy would lead to a FM magnetization along the *y*-axis, we employ an exchange bias field along the *x*-axis using Co_84_Fe_16_/Ir_22_Mn_78_ bilayers^10^,^21^,^22^,^23^,^24^,^25^,^26^ as shown in Fig. 2(a). The thicknesses of Co_84_Fe_16_ and Ir_22_Mn_78_ are 3 nm and 7 nm, respectively. To protect Co_84_Fe_16_/Ir_22_Mn_78_ bilayers, 40 nm thick Au capping layers are deposited. During the sputtering of Co_84_Fe_16_ and Ir_22_Mn_78_, we applied magnetic fields of +20 mT and −20 mT along the *x*-axis, respectively, to obtain two types of electrodes (A- and B-types) with different directions of exchange bias fields. Due to interfacial exchange interaction between the Co_84_Fe_16_ and Ir_22_Mn_78_, the first interfacial layer of Ir_22_Mn_78_ has the same magnetization direction as the Co_84_Fe_16_ layer. The antiferromagnetic order of Ir_22_Mn_78_ causes subsequent layers to have alternating magnetizations. The antiferromagnetic order is very stable, so that the ferromagnetic Co_84_Fe_16_ layer retains its magnetization direction even without a magnetic field. As shown in Fig. 2(b) the exchange fields are −11 mT and +10 mT, and at the remanent state the magnetization directions of types A and B are opposite. Therefore, by changing the applied magnetic field direction during film growth, we can set the FM in a preferred magnetization direction at zero magnetic field and realize parallel and antiparallel alignments of the two FMs.

Using FMs type A and B shown in Fig. 2(a), we can implement the P-ST and AP-ST. The realization of P-ST is relatively simple. If identical types of FMs for the source and drain electrodes (two A-type or two B-type FMs) are used, the transistor would operate as a P-ST. The AP-ST would consist of an A-type source and a B-type drain, or vice versa. The first step to accomplish AP-ST is to apply a magnetic field larger than the exchange bias field along the +*x* direction. At this moment the magnetization of both A-type and B-type electrodes are along the +*x*-axis. When the magnetic field returns to zero, the magnetization directions of A-type and B-type are along the +*x* and −*x* directions, respectively, due to the opposite sign of exchange bias as shown in Fig. 2(b).

### Parallel and antiparallel types of spin transistors

**Figure 3:**
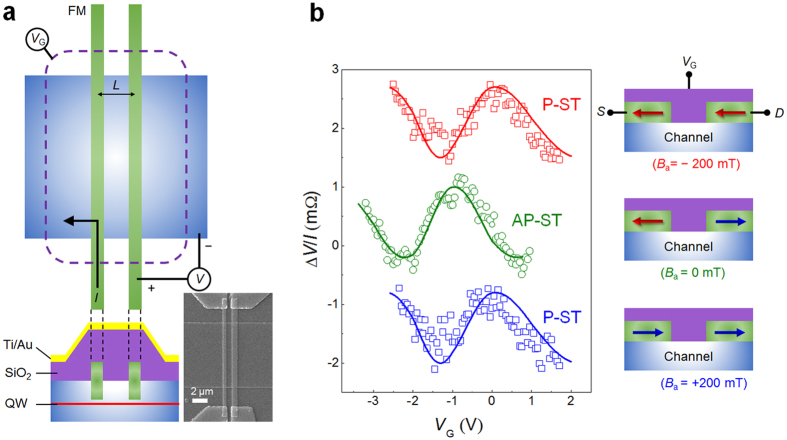
Signals of spin transistor as a function of magnetization alignment between source and drain. (**a**) Device structure. The inset is the scanning electron micrograph of the device which is taken before depositing gate electrode for clarity. (**b**) Gate control of spin FET signal. Spin transistor consists of an A-type and a B-type electrodes as a source and a drain. The channel length *L* is 1.6 μm. *I* = 1 mA, *T* = 1.8 K. The solid lines are obtained from the gate dependence of the Rashba parameter.

Figure 3(a) shows the device structure of the spin transistor which has an A-type and a B-type FMs as a source and a drain, respectively. The output voltage is determined by the spin precession angle, Δθ, which is proportional to the Rashba effective field and modulated by *V*_G_. In order to confirm the purely spin current operation, non-local measurement^18^,^27^,^28^,^29^ is utilized. With an applied magnetic field larger than the exchange bias field of the A-type source along the −*x* direction (*B*_a_ = −200 mT), the magnetization of the source is switched to the −*x* direction, so that the magnetization vectors of the source (A-type FM) and drain (B-type FM) are parallel as shown in the top right of Fig. 3(b). In the parallel alignment, the output voltage is maximum for *V*_G_ = −2.5 V and minimum for *V*_G_ = −1 V (top of Fig. 3(b)). At remanence (*B*_a_ = 0), the magnetizations of the source and drain are antiparallel, due to the opposite exchange bias field. Thus, the output voltage is maximum for *V*_G_ = −1 V and minimum for *V*_G_ = −2.5 V (middle of Fig. 3(b)). With a large magnetic field in the +*x* direction (*B*_a_ = +200 mT), the two FMs are again parallel, so the gate modulation signal (bottom of Fig. 3(b)) is the same as that at negative saturation state. The solid lines plotted in Fig. 3(b) are the expected resistance modulation, calculated using the experimental values of the Rashba parameter or spin precession angle as a function of *V*_G_ presented in Fig. 1(b), which show excellent quantitative agreement with the experimental data. These fittings assume an arbitrary phase shift of spin precession angle(Supplementary Section 1). The signals in Fig. 3 show that complementary operations with spin transistors based on the parallel and antiparallel alignments of two FMs are possible, similar to the conventional scheme consisting of *n*- and *p*-MOSs.

### Spin-based complementary logic devices

**Figure 4:**
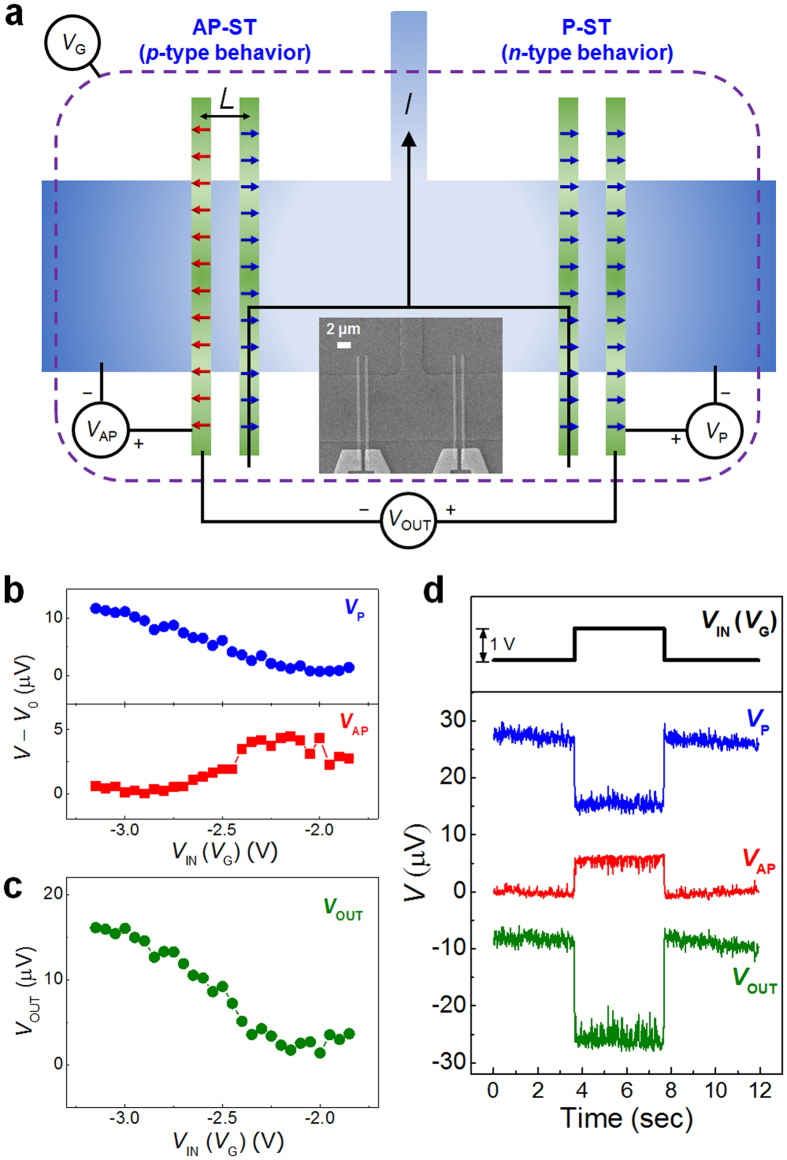
Inverter operation using complementary spin transistors. (**a**) Structure of inverter. The inset is the scanning electron micrograph of the device which is taken before depositing gate electrode for clarity. Gate dependence of output signals for (**b**) individual transistors and (**c**) inverter. (**d**) Output signals with a step input signal. *L* = 1.3 μm, *I* = 1 mA, *T* = 1.8 K. With repeating more than ten times for each logic device, successful operations are obtained for all attempts.

For real complementary logic application, both transistors (P-ST and AP-ST) are required at the same time with no applied magnetic field. To demonstrate the complementary operation, we design an inverter as shown in Fig. 4(a). The inset in Fig. 4(a) shows the scanning electron micrograph of the fabricated inverter device. The lateral size of two FMs is 0.5 μm × 15 μm. Two types of transistors are connected in serial and bias current is evenly applied to the each transistor. The measurement geometry is different from the conventional inverter because we adopt a non-local geometry. The P-ST utilizes identical types of FMs for source and drain (two A-type or two B-type FMs), whereas the AP-ST consists of A-type and B-type electrodes. After applying a magnetic field along the +*x* direction up to the saturation field and then returning to zero field, the magnetization directions of A-type and B-type electrodes are along the +*x* and −*x* directions, respectively. After setting the magnetization directions of two transistors, no external magnetic field is applied during the inverter operation. The gate voltage *V*_G_ acts as the inverter input voltage *V*_IN_, while the inverter output voltage *V*_OUT_ is the potential difference between the output voltages of the P-ST (*V*_P_) and the AP-ST (*V*_AP_).

Figure 4(b) and (c) show the output signals for the individual transistors and the inverter, respectively. When *V*_IN_ = *V*_G_ = −3 V, the P-ST is OFF, and the AP-ST is ON. Thus, *V*_OUT_, which is the potential difference between *V*_P_ and *V*_AP_ is in the “High” state. For *V*_IN_ = −2 V, only the P-ST is ON resulting in *V*_OUT_ = “Low”. We also monitored the output voltage and individual transistor signals with a step input signal. As shown in Fig. 4(d), the AP-ST is ON for *V*_IN_ = −3 V (“Low”) and the P-ST is ON for *V*_IN_ = −2 V (“High”). The input voltage is inverted at the output terminal which is the voltage difference between *V*_P_ and *V*_AP_. Thus, this device has the same function as the conventional inverter. Using a similar method as shown above, other spin based logic operations are also possible. The *p*- and *n*-MOSs can be replaced by the P-ST and the AP-ST, respectively, in the spin based logic devices.

## Discussion

In this measurement, −3 V and −2 V were selected for the low and high states, respectively, but multiple sets of operation input voltages (gate voltages) can be chosen due to the oscillatory behavior of the gate controlled spin orientation shown in Fig. 3(b). In addition, the oscillatory period can be modulated by varying the thickness of the gate oxide. In our experiments, we utilized a 100 nm thick gate oxide to exclude leakage induced side effects. The required gate voltage is usually proportional to the oxide thickness. If the gate oxide thickness is reduced to 5 nm, the operation voltage can be theoretically as small as 50 mV. By using ferromagnetic source and drains, in place of doped semiconductor regions, the diffusion capacitance is reduced, so that the power consumption is minimized in this spin logic operation.

For the transistor operation in this work, the non-local geometry is selected to exclude side effects and to confirm the pure spin operation. In real application, we had better design a local geometry (Supplementary Section 2), where the current flows into output terminal to improve the cascade property^9^. Thus, further experiments including current path modification are required for complex logic circuits. In addition, to operate the device at room temperature, we should enhance the spin injection efficiency by control of interface and develop a channel system with strong Rashba effect at a higher temperature. Usually, the channel with a higher Rashba effect has a short spin diffusion length, so nanofabrication for reducing the channel length is also required for piratical applications.

We demonstrate the spin-based complementary transistors. The complementary logic device utilizes parallel- and antiparallel-type spin transistors which play the roles of *n*- and *p*-MOS of the conventional CMOS technology. These results show the feasibility of the low power spin logic gates without a complicated doping process. Experimental demonstration of an inverter highlights the possibility of the large scale integration of spin based logic gates.

## Methods

### Device fabrication

A quantum well system for spin transistor channel^3^,^18^ was epitaxially grown in molecular beam epitaxy system. The vertical structure of the channel is InAs (2 nm)/In_0.52_Al_0.48_As (20 nm)/In_0.53_Ga_0.47_As (13.5 nm)/InAs quantum well (2 nm)/In_0.53_Ga_0.47_As (2.5 nm)/In_0.52_Al_0.48_As (6 nm)/*n*^+^ In_0.52_Al_0.48_As (7 nm)/In_0.52_Al_0.48_As buffer (300 nm)/semi-insulating InP (001) substrate. The In_0.52_Al_0.48_As and In_0.53_Ga_0.47_As cladding layers were the potential barrier to confine the electrons in the quantum well. The channel was defined using photolithography and an Ar ion milling. The patterns for ferromagnetic source and drain electrodes were defined by electron beam lithography. To adjust the interfacial resistance, parts of the cladding layers were milled out and then Co_84_Fe_16_/Ir_22_Mn_78_ bilayers were deposited using sputtering. The transistor channel and ferromagnetic electrodes were covered by a 100 ± 10 nm thick SiO_2_ with a gate electrode.

### Measurement technique

The vibrating sample magnetometer was utilized for observing magnetization curves. Gate controlled non-local measurements were performed using lock-in techniques inside a temperature controllable cryostat for probing the electrical characteristics.

## Additional Information

**How to cite this article:** Park, Y. H. *et al*. Complementary spin transistor using a quantum well channel. *Sci. Rep.* **7**, 46671; doi: 10.1038/srep46671 (2017).

**Publisher's note:** Springer Nature remains neutral with regard to jurisdictional claims in published maps and institutional affiliations.

## Supplementary Material

Supplementary Information
