# Supplementary material for: Complementary spin transistor using a quantum well channel
Source: Sci Rep. 2017 Apr 20;7:46671. doi: 10.1038/srep46671 (PMC5397970; doi:10.1038/srep46671)
Supplement: Supplementary Information [file srep46671-s1.pdf]

Supplementary Information for “Complementary spin transistor using a quantum well channel”

Youn Ho Park[1,2], Jun Woo Choi[1], Hyung-jun Kim[1], Joonyeon Chang[1], Suk Hee Han[1], Heon-Jin Choi[2], and Hyun Cheol Koo[1,3,*]

[1]Center for Spintronics, Korea Institute of Science and Technology, Seoul 02792, Korea

[2]Departement of Materials Science and Engineering, Yonsei University, Seoul 03722, Korea

[3]KU-KIST Graduate School of Converging Science and Technology, Korea University, Seoul 02481, Korea

[*]To whom correspondence should be addressed; E-mail: hckoo@kist.re.kr

Contents

Section 1. Phase shift of spin precession

Section 2. Logic simulations using conventional local geometry

Supplementary Figure 1. Spin-based NAND gate simulation using conventional local geometry.

Supplementary Figure 2. Spin-based NOR gate simulation using conventional local geometry.

Section 1. Phase shift of spin precession

The fitted solid lines are obtained from the gate controlled Rashba parameter (α) and the equations given by

$$V_{\text{P-ST}} = A\cos(\Delta\theta + \varphi) = A\cos(2m^{*}\alpha L/\hbar^{2} + \varphi) \text{ for P-ST,} \quad (1)$$

$$V_{\text{AP-ST}} = A\cos(\Delta\theta + \varphi) = A\cos(2m^{*}\alpha L/\hbar^{2} + \pi + \varphi) \text{ for AP-ST,} \quad (2)$$

where the amplitude A is assumed to be a phenomenological parameter. In these equations, φ is an arbitrary phase shift and one possible reason of this arbitrary phase shift might be due to the slightly tilted alignment of the magnetization of the source or drain.

Section 2. Logic simulations using conventional local geometry

In the main body of this manuscript, the non-local geometry is adopted to exclude side effects and to utilize the only pure spin current. However, the conventional local geometry is usually utilized for the conventional logic devices due to the cascade issue. For the logic gate using a local geometry, circuit design and current path are same as those of the conventional complementary logic gate as shown in Supplementary Figs. 1 and 2. In this section, logic calculations for local geometry are performed based on the experimental results of complementary spin transistors shown in the main body of this manuscript. The calculation results of NAND and NOR gates are illustrated in Supplementary Figs. 1 and 2. These simulation results show that our design of complementary spin transistor is also valid for the conventional local geometry.

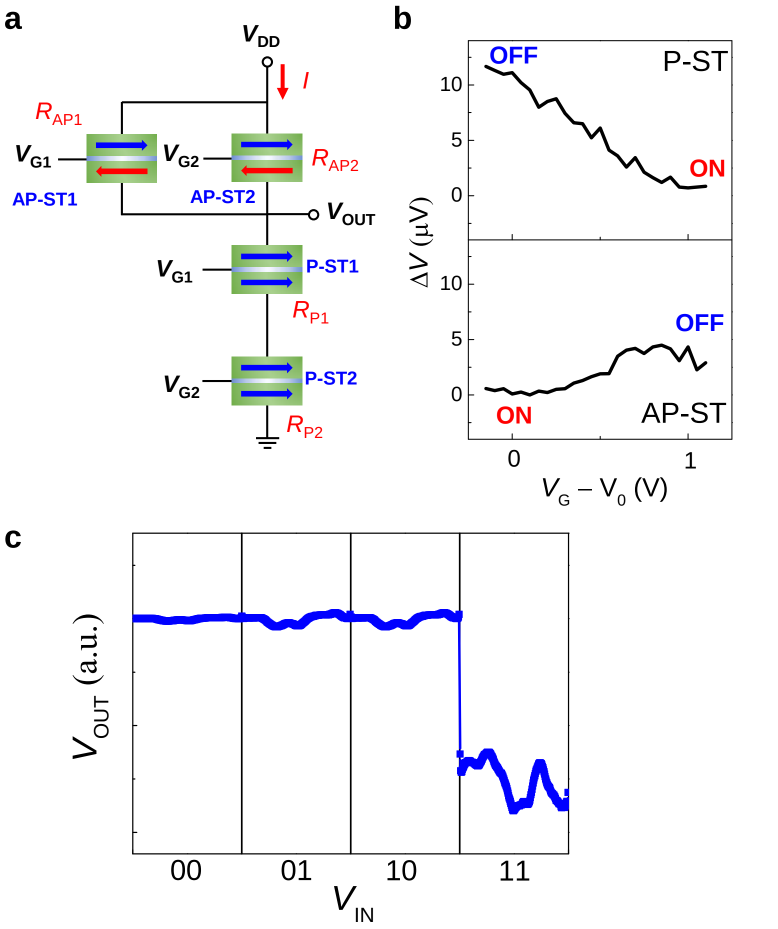

d

V_{G1}	V_{G2}	AP-ST1	AP-ST2	P-ST1	P-ST2	V_{OUT}
0	0	ON	ON	OFF	OFF	1
0	1	ON	OFF	OFF	ON	1
1	0	OFF	ON	ON	OFF	1
1	1	OFF	OFF	ON	ON	0

Supplementary Figure 1. Spin-based NAND gate simulation using conventional local geometry. (**a**) NAND gate design. (**b**) Characteristics of parallel and antiparallel types of spin transistors (P-ST and AP-ST). (**c**) Output of spin-based NAND gate. (**d**) On and off states of individual transistors and truth table. In (**c**), logic gate calculation is performed based on the experimental results of complementary spin transistors.

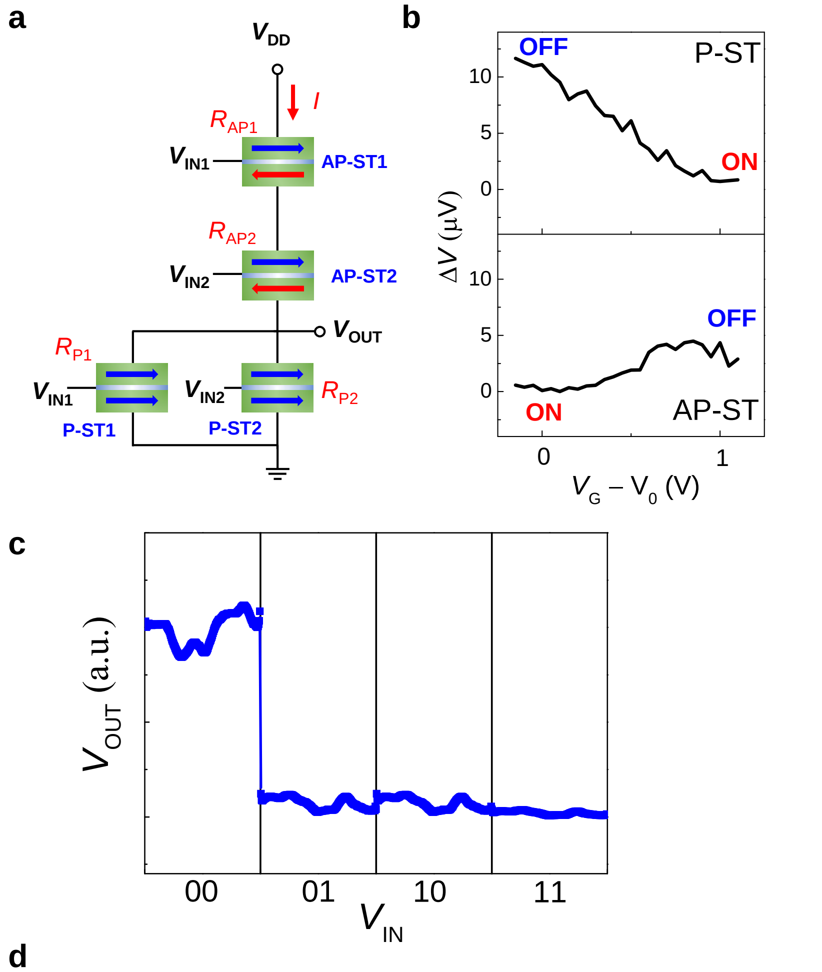

d

V_{G1}	V_{G2}	AP-ST1	AP-ST2	P-ST1	P-ST2	V_{OUT}
0	0	ON	ON	OFF	OFF	1
0	1	ON	OFF	OFF	ON	0
1	0	OFF	ON	ON	OFF	0
1	1	OFF	OFF	ON	ON	0

Supplementary Figure 2. Spin-based NOR gate simulation using conventional local geometry. (**a**) NOR gate design. (**b**) Characteristics of parallel and antiparallel types of spin transistors (P-ST and AP-ST). (**c**) Output of spin-based NOR gate. (**d**) On and off states of individual transistors and truth table. In (**c**), logic gate calculation is performed based on the experimental results of complementary spin transistors.
